# *catena*-Poly[[bis­(*p*-toluene­sulfonato-κ*O*)palladium(II)]bis­(μ-1,3-di-4-pyridylpropane-κ^2^ *N*:*N*′)]

**DOI:** 10.1107/S1600536809032760

**Published:** 2009-08-22

**Authors:** Suwen Wang, Tianyu Yang, Zhongfang Li, Xianjin Yu

**Affiliations:** a College of Chemical Engineering, Shandong University of Technology, Zibo 255049, People’s Republic of China; b The College of Life Sciences, Northwest University, Xi-an 710069, People’s Republic of China

## Abstract

In the title compound, [Pd(C_7_H_7_O_3_S)_2_(C_13_H_14_N_2_)_2_]_*n*_, the metal ion, located on a twofold rotation axis, exhibits a slightly distorted octa­hedral coordination environment, with bond angles that deviate by at most 2.2° from an ideal geometry, completed by two O atoms from two deprotonated *p*-toluene­sulfonic acid ligands and four N atoms from four 1,3-di-4-pyridylpropane ligands. One of the sulfonate O atoms is disordered over two positions [ratio 0.70 (5):0.30 (5)].

## Related literature

For the potential applications of metal-organic frameworks, see: Jia *et al.* (2007 ▶); Li *et al.* (1996 ▶); Seo *et al.* (2000 ▶); Hagrman *et al.* (1999 ▶); Yaghi *et al.* (1998 ▶); Kortz *et al.* (2003 ▶); Liu *et al.* (2007 ▶); Wang *et al.* (2007 ▶). 1,3-Di(4-pyrid­yl)propane has versatile coordination modes with transition metal centers, see: Xu *et al.* (2004 ▶); Zhu *et al.* (2002 ▶); Mock & Morsch (2001 ▶). 
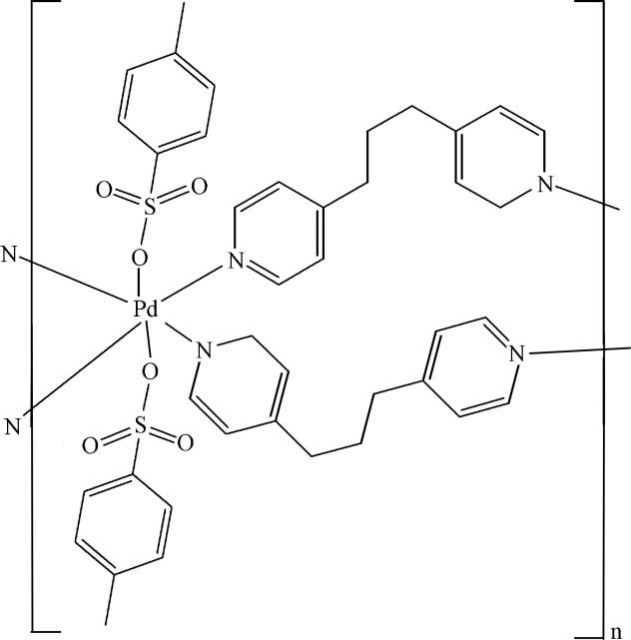


## Experimental

### 

#### Crystal data

[Pd(C_7_H_7_O_3_S)_2_(C_13_H_14_N_2_)_2_]
*M* *_r_* = 845.3
Orthorhombic, 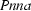

*a* = 23.818 (2) Å
*b* = 17.4359 (10) Å
*c* = 9.3341 (10) Å
*V* = 3876.3 (6) Å^3^
*Z* = 4
Mo *K*α radiation
μ = 0.64 mm^−1^
*T* = 273 K
0.12 × 0.08 × 0.01 mm

#### Data collection

Bruker APEXII CCD area-detector diffractometer
Absorption correction: multi-scan (*SADABS*; Bruker, 2001 ▶) *T*_min_ = 0.927, *T*_max_ = 0.994
18799 measured reflections
3374 independent reflections
2761 reflections with *I* > 2σ(*I*)
*R*_int_ = 0.068

#### Refinement

*R*[*F* ^2^ > 2σ(*F* ^2^)] = 0.033
*wR*(*F* ^2^) = 0.091
*S* = 1.08
3374 reflections
251 parameters
H-atom parameters not refined
Δρ_max_ = 0.61 e Å^−3^
Δρ_min_ = −0.67 e Å^−3^

### 

Data collection: *APEX2* (Bruker, 2004 ▶); cell refinement: *SAINT-Plus* (Bruker, 2001 ▶); data reduction: *SAINT-Plus*; program(s) used to solve structure: *SHELXS97* (Sheldrick, 2008 ▶); program(s) used to refine structure: *SHELXL97* (Sheldrick, 2008 ▶); molecular graphics: *SHELXTL* (Sheldrick, 2008 ▶); software used to prepare material for publication: *SHELXTL*.

## Supplementary Material

Crystal structure: contains datablocks global, I. DOI: 10.1107/S1600536809032760/bx2229sup1.cif

Structure factors: contains datablocks I. DOI: 10.1107/S1600536809032760/bx2229Isup2.hkl

Additional supplementary materials:  crystallographic information; 3D view; checkCIF report

## Figures and Tables

**Fig. 1.**
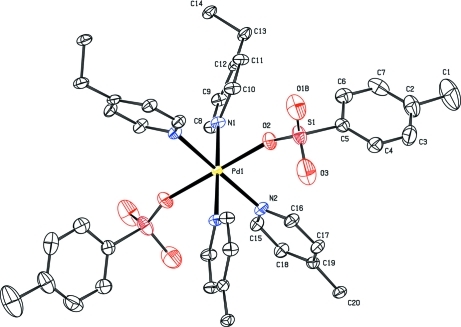
A view of (I) with the unique atom-labelling scheme. Displacement ellipsoids are drawn at the 30% probability level. Minor component of disordered O1 atom as well as H atoms have been omitted. Unlabeled atoms are related to labeled atoms by the symmetry code (x, 1/2 - y, 1/2 - z)

## supplementary crystallographic information

### Comment

Design and construction of metal-organic frameworks (MOFs) have attracted considerable attention in recent years, not only for their intriguing structural motifs but also for their potential applications in the areas of catalysis, separation, gas absorption, molecular recognition, nonlinear optics, and magnetochemistry (Jia *et al.* (2007); Li *et al.* (1996); Seo *et al.* (2000); Hagrman *et al.* (1999); Yaghi *et al.* (1998); Kortz *et al.* (2003); Liu *et al.* (2007); Wang *et al.* (2007)). A successful strategy for the design and synthesis of predictable MOFs is the assembly reaction between metal ions and well designed organic ligands. 1,3-di(4-pyridyl)proane is a very good choice for preparing such MOFs because of its versatile coordination modes with transition metal centers (Xu *et al.* (2004); Zhu *et al.* (2002); Mock *et al.* (2001)). We report here the crystal and molecular structure of the title compound, (I).

In the asymmetric unit of complex (I), exhibit one 1,3-di(4-pyridyl)propane ligand, one depronated *p*-toluenesulfonic acid, and one Pd(II) ion, figure 1.The metal exhibits an octahedral coordination environment with bond angles that do not exceed 2.2° from the ideal geometry completed by two oxygen atoms from two depronated *p*-Toluenesulfonic acid and four nitrogen atoms from four 3-(2-pyridyl)pyrazole ligand.The bond distances of Pd—O and Pd—N are in the range of 2.326 (2)–2.339 (2) and 2.338 (2) Å, respectively.The O1 atom is disordered over two positions [0.70 (5)/0.30 (5)].

### Experimental

A mixture of Pd(II) chloride (1 mmoL), *p*-Toluenesulfonic acid (1 mmoL), and 1,3-di(4-pyridyl)proane (1 mmoL) in 10 ml distilled water sealed in a 25 ml Teflon-lined stainless steel autoclave was kept at 433 K for three days. Colorless crystals suitable for the X-ray experiment were obtained. Anal. Calc. for C_40_H_42_N_4_O_6_PdS_2_: C 56.78, H 4.97, N 6.62%; Found: C 56.35, H 4.78, N 6.52%.

### Refinement

All H atoms were geometrically positioned and refined using a riding model, with C—H = 0.93 A for the aryl, 0.97 A for the methylene, and 0.96 A for the methyl H atoms. *U*_iso_(H) = 1.2Ueq(C) for the aryl, methine and methylene H atoms, and 1.5Ueq(C) for methyl H atoms. The atom O1 is disordered and was modelled using a split model with refined population parameters [O1B/O1A = 0.70 (5)/0.30 (5)].

### Crystal data

**Table:**

[Pd(C_7_H_7_O_3_S)_2_(C_13_H_14_N_2_)_2_]	*F*(000) = 1744
*M**_r_* = 845.3	*D*_x_ = 1.448 Mg m^−^^3^
Orthorhombic, *P**n**n**a*	Mo *K*α radiation, λ = 0.71073 Å
Hall symbol: -P 2a 2bc	Cell parameters from 7795 reflections
*a* = 23.818 (2) Å	θ = 2.4–28.2°
*b* = 17.4359 (10) Å	µ = 0.64 mm^−^^1^
*c* = 9.3341 (10) Å	*T* = 273 K
*V* = 3876.3 (6) Å^3^	Block, colorless
*Z* = 4	0.12 × 0.08 × 0.01 mm

### Data collection

**Table:**

Bruker APEXII CCD area-detector diffractometer	3374 independent reflections
Radiation source: fine-focus sealed tube	2761 reflections with *I* > 2σ(*I*)
graphite	*R*_int_ = 0.068
φ and ω scans	θ_max_ = 25°, θ_min_ = 2.3°
Absorption correction: multi-scan (*SADABS*; Bruker, 2001)	*h* = −28→26
*T*_min_ = 0.927, *T*_max_ = 0.994	*k* = −20→20
18799 measured reflections	*l* = −11→9

### Refinement

**Table:**

Refinement on *F*^2^	Primary atom site location: structure-invariant direct methods
Least-squares matrix: full	Secondary atom site location: difference Fourier map
*R*[*F*^2^ > 2σ(*F*^2^)] = 0.033	Hydrogen site location: inferred from neighbouring sites
*wR*(*F*^2^) = 0.091	H-atom parameters not refined
*S* = 1.08	*w* = 1/[σ^2^(*F*_o_^2^) + (0.0492*P*)^2^ + 0.7971*P*] where *P* = (*F*_o_^2^ + 2*F*_c_^2^)/3
3374 reflections	(Δ/σ)_max_ = 0.047
251 parameters	Δρ_max_ = 0.61 e Å^−^^3^
0 restraints	Δρ_min_ = −0.67 e Å^−^^3^

### Special details

**Table:**

Geometry. All s.u.'s (except the s.u. in the dihedral angle between two l.s. planes) are estimated using the full covariance matrix. The cell s.u.'s are taken into account individually in the estimation of s.u.'s in distances, angles and torsion angles; correlations between s.u.'s in cell parameters are only used when they are defined by crystal symmetry. An approximate (isotropic) treatment of cell s.u.'s is used for estimating s.u.'s involving l.s. planes.
Refinement. Refinement of *F*^2^ against ALL reflections. The weighted *R*-factor *wR* and goodness of fit *S* are based on *F*^2^, conventional *R*-factors *R* are based on *F*, with *F* set to zero for negative *F*^2^. The threshold expression of *F*^2^ > 2σ(*F*^2^) is used only for calculating *R*-factors(gt) *etc*. and is not relevant to the choice of reflections for refinement. *R*-factors based on *F*^2^ are statistically about twice as large as those based on *F*, and *R*- factors based on ALL data will be even larger.

### Fractional atomic coordinates and isotropic or equivalent isotropic displacement parameters (Å^2^)

**Table:**

	*x*	*y*	*z*	*U*_iso_*/*U*_eq_	Occ. (<1)
Pd1	0.405803 (9)	0.25	0.25	0.02547 (12)	
S1	0.38959 (4)	0.12445 (5)	0.56888 (8)	0.0569 (2)	
O1A	0.453 (3)	0.1210 (9)	0.624 (7)	0.110 (16)	0.30 (5)
O1B	0.4230 (9)	0.1297 (6)	0.6913 (16)	0.095 (4)	0.70 (5)
O2	0.40587 (9)	0.15978 (13)	0.4355 (3)	0.0623 (6)	
O3	0.33358 (13)	0.15336 (15)	0.6086 (3)	0.1029 (11)	
N1	0.47636 (8)	0.18031 (12)	0.1367 (2)	0.0352 (5)	
N2	0.33394 (8)	0.17892 (12)	0.1424 (3)	0.0383 (5)	
C1	0.3609 (3)	−0.2217 (3)	0.4820 (7)	0.144 (2)	
H1A	0.3825	−0.2462	0.5559	0.216*	
H1B	0.3744	−0.2378	0.3899	0.216*	
H1C	0.3221	−0.2357	0.4918	0.216*	
C2	0.3669 (2)	−0.1344 (2)	0.4956 (4)	0.0741 (11)	
C3	0.32559 (17)	−0.0900 (2)	0.5550 (4)	0.0739 (11)	
H3	0.2924	−0.1132	0.5847	0.089*	
C4	0.33176 (13)	−0.01173 (19)	0.5722 (3)	0.0561 (8)	
H4	0.3027	0.0166	0.6125	0.067*	
C5	0.37985 (12)	0.02464 (16)	0.5309 (3)	0.0405 (6)	
C6	0.42192 (14)	−0.0190 (2)	0.4681 (3)	0.0520 (8)	
H6	0.4549	0.0043	0.4371	0.062*	
C7	0.41461 (17)	−0.0978 (2)	0.4516 (4)	0.0663 (10)	
H7	0.4431	−0.1264	0.4093	0.08*	
C8	0.48162 (10)	0.16862 (16)	−0.0055 (3)	0.0386 (6)	
H8	0.4599	0.1982	−0.067	0.046*	
C9	0.51733 (10)	0.11541 (16)	−0.0644 (3)	0.0389 (6)	
H9	0.519	0.1092	−0.1632	0.047*	
C10	0.50992 (12)	0.13828 (18)	0.2226 (3)	0.0408 (7)	
H10	0.5079	0.1461	0.321	0.049*	
C11	0.54674 (10)	0.08471 (16)	0.1715 (3)	0.0410 (7)	
H11	0.5691	0.0572	0.2351	0.049*	
C12	0.55099 (10)	0.07100 (14)	0.0245 (3)	0.0339 (6)	
C13	0.58962 (10)	0.01068 (17)	−0.0364 (4)	0.0442 (7)	
H13A	0.5701	−0.0172	−0.1113	0.053*	
H13B	0.5993	−0.0256	0.0385	0.053*	
C14	0.64305 (10)	0.04495 (15)	−0.0976 (3)	0.0398 (6)	
H14A	0.662	0.0064	−0.1547	0.048*	
H14B	0.6332	0.0871	−0.1605	0.048*	
C15	0.32321 (11)	0.17269 (16)	0.0008 (3)	0.0402 (6)	
H15	0.3494	0.1923	−0.0634	0.048*	
C16	0.29563 (12)	0.15034 (19)	0.2325 (3)	0.0423 (7)	
H16	0.3019	0.1544	0.3306	0.051*	
C17	0.24731 (11)	0.11523 (15)	0.1860 (3)	0.0413 (6)	
H17	0.2221	0.0952	0.2523	0.05*	
C18	0.27591 (11)	0.13906 (16)	−0.0530 (3)	0.0419 (7)	
H18	0.2705	0.136	−0.1516	0.05*	
C19	0.23581 (10)	0.10944 (14)	0.0402 (3)	0.0341 (6)	
C20	0.18311 (10)	0.07391 (16)	−0.0165 (3)	0.0380 (6)	
H20A	0.1932	0.0314	−0.0783	0.046*	
H20B	0.1637	0.1115	−0.0748	0.046*	

### Atomic displacement parameters (Å^2^)

**Table:**

	*U*^11^	*U*^22^	*U*^33^	*U*^12^	*U*^13^	*U*^23^
Pd1	0.01639 (17)	0.02857 (17)	0.03145 (18)	0	0	−0.00186 (10)
S1	0.0873 (6)	0.0455 (4)	0.0379 (4)	−0.0102 (4)	−0.0087 (4)	0.0075 (3)
O1A	0.17 (3)	0.066 (7)	0.09 (3)	−0.003 (9)	−0.09 (2)	0.001 (8)
O1B	0.157 (9)	0.075 (3)	0.051 (5)	−0.031 (4)	−0.053 (6)	−0.004 (4)
O2	0.0648 (14)	0.0570 (14)	0.0652 (15)	−0.0050 (10)	0.0055 (11)	0.0284 (12)
O3	0.131 (3)	0.0669 (17)	0.111 (2)	0.0160 (17)	0.070 (2)	−0.0008 (16)
N1	0.0283 (11)	0.0425 (12)	0.0347 (12)	0.0029 (9)	−0.0016 (9)	−0.0028 (10)
N2	0.0273 (11)	0.0437 (13)	0.0438 (14)	−0.0045 (9)	0.0016 (10)	−0.0048 (11)
C1	0.238 (7)	0.055 (3)	0.139 (5)	−0.016 (4)	−0.024 (5)	−0.015 (3)
C2	0.112 (3)	0.050 (2)	0.060 (2)	−0.011 (2)	−0.016 (2)	0.0012 (18)
C3	0.074 (2)	0.067 (2)	0.081 (3)	−0.027 (2)	−0.007 (2)	0.012 (2)
C4	0.0464 (17)	0.061 (2)	0.061 (2)	−0.0019 (15)	0.0018 (15)	0.0076 (17)
C5	0.0437 (16)	0.0474 (16)	0.0304 (14)	0.0013 (13)	−0.0044 (12)	0.0069 (12)
C6	0.0503 (17)	0.070 (2)	0.0361 (16)	0.0020 (16)	−0.0016 (14)	0.0063 (15)
C7	0.092 (3)	0.065 (2)	0.0421 (19)	0.029 (2)	−0.0071 (19)	−0.0055 (17)
C8	0.0314 (14)	0.0502 (16)	0.0342 (14)	0.0048 (12)	0.0004 (12)	0.0043 (12)
C9	0.0332 (14)	0.0546 (16)	0.0291 (14)	0.0025 (12)	0.0021 (11)	−0.0025 (12)
C10	0.0342 (15)	0.0593 (18)	0.0290 (14)	0.0092 (14)	−0.0006 (11)	−0.0013 (12)
C11	0.0300 (14)	0.0534 (17)	0.0397 (17)	0.0118 (12)	−0.0016 (12)	0.0052 (13)
C12	0.0199 (12)	0.0379 (14)	0.0438 (16)	−0.0053 (10)	0.0046 (11)	−0.0043 (12)
C13	0.0272 (14)	0.0428 (16)	0.063 (2)	−0.0035 (11)	0.0099 (13)	−0.0097 (15)
C14	0.0245 (13)	0.0457 (16)	0.0491 (17)	−0.0017 (11)	0.0087 (12)	−0.0088 (13)
C15	0.0341 (14)	0.0463 (16)	0.0402 (16)	−0.0064 (12)	0.0046 (12)	−0.0023 (13)
C16	0.0328 (15)	0.0582 (19)	0.0359 (16)	−0.0070 (14)	−0.0038 (12)	0.0000 (13)
C17	0.0299 (14)	0.0512 (17)	0.0430 (16)	−0.0085 (12)	−0.0003 (13)	0.0016 (14)
C18	0.0399 (15)	0.0504 (17)	0.0353 (15)	−0.0053 (13)	−0.0007 (12)	−0.0038 (13)
C19	0.0298 (13)	0.0329 (13)	0.0395 (15)	0.0017 (10)	−0.0011 (11)	−0.0022 (11)
C20	0.0276 (14)	0.0416 (15)	0.0448 (16)	−0.0018 (11)	−0.0062 (12)	−0.0023 (13)

### Geometric parameters (Å, °)

**Table:**

Pd1—N1^i^	2.328 (2)	C7—H7	0.93
Pd1—N1	2.328 (2)	C8—C9	1.373 (4)
Pd1—O2	2.339 (2)	C8—H8	0.93
Pd1—O2^i^	2.339 (2)	C9—C12	1.389 (4)
Pd1—N2	2.340 (2)	C9—H9	0.93
Pd1—N2^i^	2.340 (2)	C10—C11	1.367 (4)
S1—O1B	1.396 (6)	C10—H10	0.93
S1—O2	1.442 (2)	C11—C12	1.397 (4)
S1—O3	1.473 (3)	C11—H11	0.93
S1—O1A	1.59 (4)	C12—C13	1.508 (4)
S1—C5	1.791 (3)	C13—C14	1.517 (3)
N1—C8	1.348 (3)	C13—H13A	0.97
N1—C10	1.349 (3)	C13—H13B	0.97
N2—C16	1.338 (3)	C14—C20^ii^	1.516 (4)
N2—C15	1.350 (4)	C14—H14A	0.97
C1—C2	1.535 (5)	C14—H14B	0.97
C1—H1A	0.96	C15—C18	1.366 (4)
C1—H1B	0.96	C15—H15	0.93
C1—H1C	0.96	C16—C17	1.374 (4)
C2—C7	1.367 (5)	C16—H16	0.93
C2—C3	1.369 (6)	C17—C19	1.392 (4)
C3—C4	1.382 (5)	C17—H17	0.93
C3—H3	0.93	C18—C19	1.392 (4)
C4—C5	1.365 (4)	C18—H18	0.93
C4—H4	0.93	C19—C20	1.496 (3)
C5—C6	1.388 (4)	C20—C14^iii^	1.516 (4)
C6—C7	1.394 (5)	C20—H20A	0.97
C6—H6	0.93	C20—H20B	0.97
			
N1^i^—Pd1—N1	87.57 (10)	C2—C7—C6	122.1 (3)
N1^i^—Pd1—O2	90.82 (8)	C2—C7—H7	119
N1—Pd1—O2	89.13 (8)	C6—C7—H7	119
N1^i^—Pd1—O2^i^	89.13 (8)	N1—C8—C9	123.6 (3)
N1—Pd1—O2^i^	90.82 (8)	N1—C8—H8	118.2
O2—Pd1—O2^i^	179.93 (11)	C9—C8—H8	118.2
N1^i^—Pd1—N2	178.41 (7)	C8—C9—C12	119.7 (3)
N1—Pd1—N2	93.24 (8)	C8—C9—H9	120.2
O2—Pd1—N2	87.83 (8)	C12—C9—H9	120.2
O2^i^—Pd1—N2	92.23 (8)	N1—C10—C11	123.0 (2)
N1^i^—Pd1—N2^i^	93.24 (8)	N1—C10—H10	118.5
N1—Pd1—N2^i^	178.41 (7)	C11—C10—H10	118.5
O2—Pd1—N2^i^	92.23 (8)	C10—C11—C12	120.4 (2)
O2^i^—Pd1—N2^i^	87.83 (8)	C10—C11—H11	119.8
N2—Pd1—N2^i^	85.97 (10)	C12—C11—H11	119.8
O1B—S1—O2	121.7 (8)	C9—C12—C11	116.7 (2)
O1B—S1—O3	106.7 (11)	C9—C12—C13	121.1 (3)
O2—S1—O3	108.33 (16)	C11—C12—C13	122.2 (3)
O2—S1—O1A	92 (2)	C12—C13—C14	112.3 (2)
O3—S1—O1A	142 (2)	C12—C13—H13A	109.2
O1B—S1—C5	107.4 (4)	C14—C13—H13A	109.2
O2—S1—C5	106.20 (14)	C12—C13—H13B	109.2
O3—S1—C5	105.37 (15)	C14—C13—H13B	109.2
O1A—S1—C5	98.6 (8)	H13A—C13—H13B	107.9
S1—O2—Pd1	157.82 (15)	C20^ii^—C14—C13	113.3 (2)
C8—N1—C10	116.6 (2)	C20^ii^—C14—H14A	108.9
C8—N1—Pd1	126.37 (17)	C13—C14—H14A	108.9
C10—N1—Pd1	116.20 (17)	C20^ii^—C14—H14B	108.9
C16—N2—C15	117.2 (2)	C13—C14—H14B	108.9
C16—N2—Pd1	115.22 (18)	H14A—C14—H14B	107.7
C15—N2—Pd1	126.96 (18)	N2—C15—C18	123.4 (3)
C2—C1—H1A	109.5	N2—C15—H15	118.3
C2—C1—H1B	109.5	C18—C15—H15	118.3
H1A—C1—H1B	109.5	N2—C16—C17	122.6 (3)
C2—C1—H1C	109.5	N2—C16—H16	118.7
H1A—C1—H1C	109.5	C17—C16—H16	118.7
H1B—C1—H1C	109.5	C16—C17—C19	120.4 (3)
C7—C2—C3	117.1 (3)	C16—C17—H17	119.8
C7—C2—C1	121.0 (5)	C19—C17—H17	119.8
C3—C2—C1	121.9 (5)	C15—C18—C19	119.7 (3)
C2—C3—C4	121.9 (3)	C15—C18—H18	120.2
C2—C3—H3	119	C19—C18—H18	120.2
C4—C3—H3	119	C17—C19—C18	116.7 (2)
C5—C4—C3	121.0 (3)	C17—C19—C20	122.7 (2)
C5—C4—H4	119.5	C18—C19—C20	120.5 (2)
C3—C4—H4	119.5	C19—C20—C14^iii^	114.7 (2)
C4—C5—C6	118.1 (3)	C19—C20—H20A	108.6
C4—C5—S1	120.3 (2)	C14^iii^—C20—H20A	108.6
C6—C5—S1	121.5 (2)	C19—C20—H20B	108.6
C5—C6—C7	119.8 (3)	C14^iii^—C20—H20B	108.6
C5—C6—H6	120.1	H20A—C20—H20B	107.6
C7—C6—H6	120.1		
			
O1B—S1—O2—Pd1	−103.6 (11)	O3—S1—C5—C6	169.7 (2)
O3—S1—O2—Pd1	20.5 (5)	O1A—S1—C5—C6	−40 (3)
O1A—S1—O2—Pd1	−127.1 (14)	C4—C5—C6—C7	−1.3 (4)
C5—S1—O2—Pd1	133.3 (4)	S1—C5—C6—C7	174.4 (2)
N1^i^—Pd1—O2—S1	91.8 (4)	C3—C2—C7—C6	1.1 (5)
N1—Pd1—O2—S1	179.3 (4)	C1—C2—C7—C6	−177.4 (4)
N2—Pd1—O2—S1	−87.4 (4)	C5—C6—C7—C2	0.0 (5)
N2^i^—Pd1—O2—S1	−1.5 (4)	C10—N1—C8—C9	2.2 (4)
N1^i^—Pd1—N1—C8	−127.0 (2)	Pd1—N1—C8—C9	−166.94 (19)
O2—Pd1—N1—C8	142.2 (2)	N1—C8—C9—C12	−0.9 (4)
O2^i^—Pd1—N1—C8	−37.9 (2)	C8—N1—C10—C11	−1.7 (4)
O2—Pd1—N1—C10	−27.1 (2)	Pd1—N1—C10—C11	168.6 (2)
O2^i^—Pd1—N1—C10	152.9 (2)	N1—C10—C11—C12	−0.2 (4)
N2—Pd1—N1—C10	−114.83 (19)	C8—C9—C12—C11	−1.0 (4)
N1—Pd1—N2—C16	128.3 (2)	C8—C9—C12—C13	178.5 (2)
O2^i^—Pd1—N2—C16	−140.8 (2)	C10—C11—C12—C9	1.5 (4)
N2^i^—Pd1—N2—C16	−53.09 (18)	C10—C11—C12—C13	−178.0 (3)
N1—Pd1—N2—C15	−61.2 (2)	C9—C12—C13—C14	78.7 (3)
O2—Pd1—N2—C15	−150.2 (2)	C11—C12—C13—C14	−101.9 (3)
O2^i^—Pd1—N2—C15	29.7 (2)	C12—C13—C14—C20^ii^	72.0 (3)
N2^i^—Pd1—N2—C15	117.4 (2)	C16—N2—C15—C18	−0.1 (4)
C7—C2—C3—C4	−0.9 (6)	Pd1—N2—C15—C18	−170.4 (2)
C1—C2—C3—C4	177.5 (4)	C15—N2—C16—C17	0.6 (4)
C2—C3—C4—C5	−0.4 (6)	Pd1—N2—C16—C17	172.0 (2)
C3—C4—C5—C6	1.5 (5)	N2—C16—C17—C19	−1.3 (5)
C3—C4—C5—S1	−174.3 (3)	N2—C15—C18—C19	0.3 (4)
O1B—S1—C5—C4	98.9 (12)	C16—C17—C19—C18	1.4 (4)
O2—S1—C5—C4	−129.5 (2)	C16—C17—C19—C20	−178.2 (3)
O3—S1—C5—C4	−14.7 (3)	C15—C18—C19—C17	−0.9 (4)
O1A—S1—C5—C4	136 (3)	C15—C18—C19—C20	178.6 (2)
O1B—S1—C5—C6	−76.7 (12)	C17—C19—C20—C14^iii^	0.2 (4)
O2—S1—C5—C6	54.9 (3)	C18—C19—C20—C14^iii^	−179.3 (2)

Symmetry codes: (i) *x*, −*y*+1/2, −*z*+1/2; (ii) *x*+1/2, *y*, −*z*; (iii) *x*−1/2, *y*, −*z*.
